# Supplementary material for: Chromothripsis during telomere crisis is independent of NHEJ, and consistent with a replicative origin
Source: Genome Res. 2019 May;29(5):737–49. doi: 10.1101/gr.240705.118 (PMC6499312; doi:10.1101/gr.240705.118)
Supplement: Supplemental Material [file supp_gr.240705.118_Supplemental_file_1.zip › contigs/annotated_contigs/DB55/contig.2.DB55_length_603_mean_cov_6.80597014925.docx]

**DB55_length_603_mean_cov_6.80597014925**

```
AATTGACACCCTAATATCACAATTAAAAGAACTAGAGAAGCAAGAGCAAACACATTCAAAACCTAGCAGAAGGCAAGAAATAACTAAGA
 >chr6:149182510-149182737 - E=7e-125
TCAGAACAGAACCGAAGGAGACAGAGACACAAAAAAACCTTAAAAAAATCAATGAATCCAGGAGCTGGTTTTTTGAAAAGATCAACAAA

ATTGATAGACCGCTAGCAAGACTAATAAAGAAGAAAAGAGAGAAGAAT|C|CATCCCATCTAGGAAGTGAGGAGCGCCTCTTCCCGGCC
                                                 >chr6:148500065-148500443 - E=5e-208
GCCATCCCATCTAGGAAGTGAGGAGCGCCTCTTCCCGGCCACCCATCATCTGAGATGTGGGGAGCACCTCTGCCCTGCCGCCCCGTCCG

GGATGTGAGGAGCGTCTCTGCCCGGCCGCCCCCTCTGAGAAGTGAGGAGACCCTCTGCCTGGCAACCGCCCCGTCTGAGAAGTGAGGAG

CCCCTCCGCCCAGCAGCCACCCCGTCTGGGAAGTGAGGAGCGTCTCTGCCCGGCAGCCACCTCGTCCGGCAGGGAGGTGGGGGGTCAGC

CCCCCGCCCGGCCAGCCACCCCGTCCGGGAGGGAGGTGGGGGGATCCGCCCCCCGCCCGGCCAGCCGCCCC
```
